# Beyond Residence: A Mobility-based Approach for Improved Evaluation of Human Exposure to Environmental Hazards

**DOI:** 10.1021/acs.est.3c04691

**Published:** 2023-10-04

**Authors:** Zhewei Liu, Chenyue Liu, Ali Mostafavi

**Affiliations:** UrbanResilience.AI Lab, Zachry Department of Civil and Environmental Engineering, Texas A&M University, College Station, Texas 77843, United States

**Keywords:** environmental hazard exposure, human mobility, environmental justice, mobility-based analysis, hazard exposure disparity

## Abstract

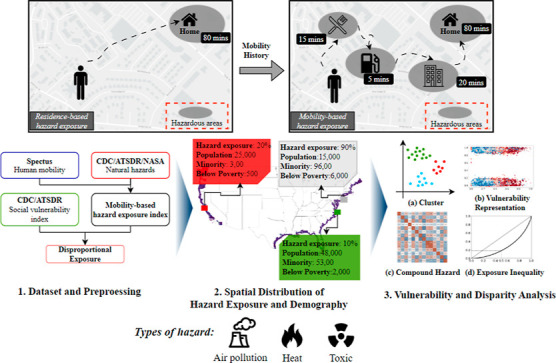

Standard environmental hazard exposure assessment methods have been primarily based on residential places, neglecting individuals’ hazard exposures due to activities outside home neighborhood and underestimating peoples’ overall hazard exposures. To address this limitation, this study proposes a novel mobility-based index for the hazard exposure evaluation. Using large-scale human mobility data, we quantify the extent of population dwell time in high environmental hazard places in 239 US counties for three environmental hazards. We explore how human mobility extends the reach of environmental hazards and leads to the emergence of latent exposure for populations living outside high-hazard areas. Notably, neglect of mobility can lead to over 10% underestimation of hazard exposures. The interplay of spatial clustering in high-hazard regions and human movement trends creates “environmental hazard traps.” Poor and ethnic minority residents disproportionately face multiple types of environmental hazards. This data-driven evidence supports the severity of these injustices. We also studied latent exposure arising from visits outside residents’ home areas, revealing millions of the population having 5 to 10% of daily activities occur in high-exposure zones. Despite living in perceived safe areas, human mobility could expose millions of residents to different hazards. These findings provide crucial insights for targeted policies to mitigate these severe environmental injustices.

## Abstract 2

This study reveals the importance of including individuals’ mobility for the accurate assessment of hazard exposure (i.e., air pollution, toxic sites, and heat).

## 1 Introduction

Environmental hazards, such as air pollution, toxic exposures, and heat, have become pressing concerns in the face of rapid urbanization, industrialization, and climate change. The adverse effects of hazard exposures on populations worldwide are increasingly recognized, with an estimated 23% of global deaths, or roughly 12.6 million deaths per year, attributable to environmental factors.^1,2^ Global climate change further aggravates the detrimental health consequences of environmental hazard exposure, underscoring the importance of accurate hazard monitoring and evaluation.^3−5^

Evaluating and monitoring environmental hazards and their impact on human populations have been the subject of numerous studies in recent years. Traditional methods for assessing hazards and populations exposure have been primarily focused on the place of residence, using empirical models, environmental data from sensors, and spatial analysis techniques to estimate hazard concentrations in different areas of a city.^6−9^ Researchers have proposed various indicators to quantify the extent of environmental hazards and risks of communities. For instance, researchers have estimated PM2.5 exposure by assigning emissions from industrial facilities to nearby census block groups and then incorporating this pollutant into calculations of the burden on racial groups and poverty status according to their residence locations.^10,11^ Likewise, heat vulnerability indices (HVI) have been developed to assess heat risk at specific locations based on the populations’ social–economic factors, land cover type, and green space.^12^ These indices reveal populations at higher exposure locations experiencing greater mortality rates during periods of high temperatures.^13,14^

However, the existing approaches tend to overlook the effects of daily activity and movement patterns, which can significantly alter an individual’s exposure to various hazards. The consideration of a mobility-based evaluation of hazard exposure provides a deeper understanding of the extent to which individuals and communities are exposed to different types of environmental hazards. The traditional residence-based methods for assessing hazard exposure do not adequately account for the fact that people’s daily activities involve movements between different locations with varying levels of hazard exposure.^15,16^ For instance, daily commuting patterns can lead to increased exposure to air pollution, even for individuals who reside in areas with relatively low levels of pollution.^17^ Similarly, exposure to urban heat can vary significantly throughout the day due to differences in land use, building materials, and the presence of green spaces, which can impact people who frequently visit parks, retail establishments, or attend sporting events.^7,18^ Lack of consideration of exposures caused by human mobility has created blind spots and also undermines the accuracy of hazard exposure assessments.

Another important aspect of hazard exposure research is the environment justice issue, which emphasizes that certain population groups and communities may be disproportionately exposed to the adverse effects of environmental hazards.^19^ Studies have shown that minority and low-income communities often face a higher burden of hazard exposure, such as air pollution and extreme heat, resulting in health disparities and social inequity.^20^ Research on air pollution exposure reveals that racial and ethnic minorities and low-income individuals are more likely to live in areas with higher concentrations of PM2.5 emissions, leading to increased health risks.^21^ Similarly, low-income and minority communities are often more susceptible to the impacts of heatwaves due to the lack of access to green spaces, inadequate housing, and limited resources for adaptation.^22^

Similar to the standard environmental hazard evaluations, the majority of environmental justice studies have mainly focused on residence-based analyses of hazard exposure disparities.^23^ However, with the recognition of the importance of human mobility in determining the extent of hazard exposure in populations, there is an increasing need to explore environmental justice issues through this lens. The fine-scaled human mobility data sets enable tracking daily activities at individual and crowd levels,^24−26^ as well as reveal the urban structures.^27−30^ By capturing the dynamic nature of the daily activities and movements, mobility-based evaluations can provide a more accurate quantification of the disparities in hazard exposure experienced by vulnerable populations.^31,32^ An example is that low-income workers who commute to industrial sites for work may face higher exposure to air pollution, even if they reside in areas with relatively low pollution levels.^17^ In addition, individuals who work in urban areas with limited green spaces may experience increased vulnerability to heat stress during the day. Integrating mobility-based evaluations into environmental justice research can deepen the understanding of the extent of latent exposure in these previously overlooked vulnerable communities, informing targeted interventions and policies to reduce disproportionate hazard exposure and its associated health and social impacts.

This study introduces a novel mobility-based index for hazard evaluation. Focusing on the US coastal areas as study regions, we assessed the impact of three significant natural hazards (i.e., air pollution, heat, and toxic sites) within these regions. Accordingly, this study aims to answer the following interrelated research questions:

- RQ1: To what extent do individuals’ mobility increase their daily hazard exposure, in addition to the exposure at their residence?
- RQ2: How much population face latent hazard exposure due to their daily mobility, despite living in no-exposure areas? Which hazards pose the greatest latent threat to the populations?
- RQ3: How do hazards differentially impact various geographic regions and diverse demographic groups? How effective is a mobility-based approach in revealing underidentified communities and exposing emerging environmental issues?

## 2 Data Set and Methodology

**Figure 1:**
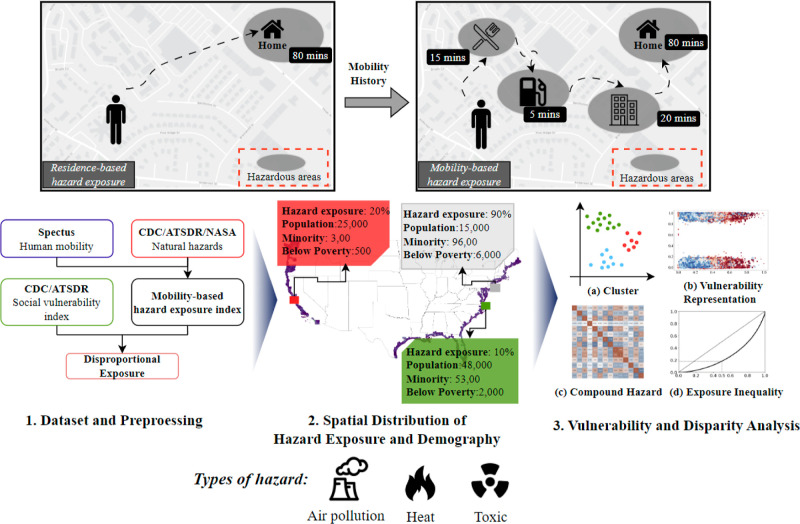
Overview of the study workflow. Human mobility data sets are combined with environmental hazard data sets to calculate mobility-based hazard exposure index based on user dwell-times in high-hazard areas. Demographic analysis is conducted to quantify the threats of hazard exposures to different subpopulations across the study regions. Third, by using clustering, correlation, and inequality analyses, the vulnerability of different communities to hazard exposures and the corresponding disparity issues are revealed.

The workflow of this study is presented in Figure 1. First, human mobility data sets are overlaid with the distributions of the targeted hazards to quantify the total duration/time that individuals spend (termed as “dwell time” throughout the paper) in high-hazard areas and nonhigh-hazard areas, and then aggregate the dwell-time exposures in creating the mobility-based hazard exposure index. We calculate the mobility-based exposure index for each hazard type at the census tract level across different US coastal counties. Second, the index is examined to reveal the extent to which human mobility extends environmental hazard exposures. Also, the mobility-based exposure index is analyzed in conjunction with demographic data to identify disparities in hazard exposure across the study regions. Third, we investigate the spatial distribution patterns and disparity in mobility-based exposure for various hazards, along with their corresponding correlations and inequalities among different subpopulations.

### 2.1 Data Sets

We selected the coastal areas in the United States as the study regions, covering 239 counties in total. The socioeconomic and hazard exposure data are collected and analyzed at the census tract level. The details related to each data set are presented below.

#### 2.1.1 Human Mobility Data Sets

The human mobility data sets are used for tracking individuals’ daily movement. In this study, the data sets were provided by Spectus Inc.,^33^ which includes the anonymized location of mobile phones and smartphone devices. Spectus compiles large-scale anonymous location information from nearly 70 million mobile devices in the United States, all gathered within a compliant framework when users consent to the location services of affiliated apps. Statistically, Spectus’s data collection extends to approximately one in four smartphones in the country, representing close to 20% of the total population.^34^ The data sets were collected in accordance with privacy practices, ensuring the collection of anonymous and privacy-compliant location data. The data set utilized in this study pertains to the entire month of April 2019; each record in the database includes an individual’s visit history
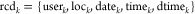
1where, rcd_*k*_ is a record in our database, user_*k*_ is a logged individual in the data set, loc_*k*_ is the location of a stop by user_*k*_, and date_*k*_, time_*k*_, dtime_*k*_ are date, time and dwell time, respectively, at the stop. The home location of each individual user_*k*_ home is inferred in accordance with previous works.^17^

#### 2.1.2 Demographic Data Sets and Social Vulnerability Index

Demographic data at census tract level are collected from the Social Vulnerability Index^35^ published by The Centers for Disease Control and Prevention’s Agency for Toxic Substances and Disease Registry (CDC ATSDR). The data covers the demographic statistics such as population, average income, and also the indicators for assessing a community’s potential susceptibility to hazards, such as percentage of ethnic minority, poverty, lack of access to transportation, and crowded housing.

#### 2.1.3 Hazard Exposure Data Sets

Our study necessitates the identification of high-hazard exposure areas, for which we consider three primary environmental hazard types: air pollution, proximity to toxic sites, and extreme heat.

For air pollution, the data sets are collected from the Environmental Justice Index,^36^ which includes the percentile rank of annual mean days exceeding the PM2.5 regulatory standard, averaged over 3 years at the census tract level. Those tracts that rank above the 50th percentile will be specified as high air pollution exposure areas.

For toxic exposure, the data specifies the percentile rank of the proportion of a tract’s area located within a 1-mile buffer of an EPA Toxic Release Inventory, again collected from the Environmental Justice Index.^36^ Similarly, those tracts that rank above the 50th percentile will be identified as areas with toxic air pollution toxic sites. The toxic sites mainly include larger facilities involved in manufacturing, metal mining, electric power generation, chemical manufacturing, and hazardous waste treatment. Until 2020, the chemical list consists of 767 individually listed chemicals and 33 chemical categories.^37,38^

For extreme heat exposure, the data are provided by the North American Land Data Assimilation System^39^ at the census tract level, which represents the number of extreme heat days occurring between May and September 2019. For each county, we employed quartiles to delineate four classes, subsequently classifying the top quartile, or 25%, of census tracts as high heat exposure areas.

### 2.2 Calculating Mobility-Based Hazard Exposure Index

The calculated mobility-based hazard exposure index at the census tract level represents an individuals’ hazard exposure with consideration of both their residence location and daily dwell time in high-hazard areas.

First, for census tract ct_*i*_, the parameter total dwell time TDT_*i*_ is computed by summing the dwell time of each visit made by individuals who reside within the tract
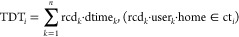
2

Then, the parameter dwell time in hazards TDT_*k*_ is defined as the sum of the dwell times recorded at stop points located within the high-hazard exposure areas (see Section 2.1)
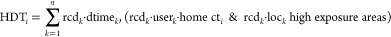
3

Finally, for a census tract ct_*i*_, the mobility-based hazard exposure index MEI^*i*^ is calculated by
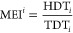
4

We calculate the tract-level MEI^*i*^ for all the three hazard types, and consequently calculate the mobility-based hazard exposure index (MEI_ap_^*i*^, MEI_t_^*i*^, and MEI_h_^*i*^) for air pollution, toxic site exposures, and extreme heat, respectively. The index captures the extent to which users residing in a census tract spend time by visiting places in high-hazard areas as a proportion of total time they spent in all places. The index range is from 0 to 1. The greater the index value, the greater the dwell in high-hazard areas and the greater the exposure.

### 2.3 Classifying the Regions Based on Mobility-Based Hazard Exposures

To identify the patterns of hazard exposures and the compounded environmental pressures across regions, a density-based clustering method (DBSCAN) was applied to classify the study regions into different categories.^40^ DBSCAN is robust to data noise and can detect clusters with arbitrary shapes and sizes without assuming spherical clusters and a specified number of clusters.

Given a data set *X* consisting of *n* data points, DBSCAN defines a neighborhood around each data point *x*_*i*_ using a distance metric *d*(.,.) and a radius ε > 0
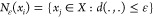
5where *N*_ε_(*x*_*i*_) is the set of points within the ε-neighborhood of *x*_*i*_. If the number of points inside this radius meets or exceeds a predetermined minimum threshold value, the point is deemed a core point and all neighboring points within the radius are incorporated into its cluster. The clusters are expanded recursively by incorporating neighboring core points and their related border points. This procedure continues until all points are either assigned to a cluster or designated as noise points if they do not belong to any cluster.

In this study, each data point *x*_*i*_ is a three-dimensional vector indicating a census tract’s exposures to three kinds of hazards, that is, *x*_*i*_ = (MEI_ap_^*i*^, MEI_t_^*i*^, and MEI_h_^*i*^), and the distance metric *d*(.,.) is defined as the Euclidean distance between the data points.

### 2.4 Hazard Exposure Disparity for Socially Vulnerable Populations

We conducted the *t*-test to examine the presence of disparities in the extent of mobility-based hazard exposure for socially vulnerable populations. *t*-Test is a statistical hypothesis to determine whether there is significant difference between the means of two groups of data. It is calculated by taking the difference between the means of the two groups and dividing it by the standard error of the difference between the means. In this study, the proportions of vulnerable subgroups (i.e., minority, below poverty, Section 2.1) among different hazard exposure regions are quantified, and the *t*-test is then applied for statistical comparisons.

## 3 Results

### 3.1 Patterns of Hazard Exposures

In this study, the regions with hazard exposures [i.e., mobility-based exposure index (MEI) > 0] are categorized into the following two classes:

- “Direct exposure regions”: the regions per se have high levels of environmental hazards, and therefore those residents living within the areas have direct exposures to the environmental hazards.
- “Latent exposure regions”: the regions that do not have environmental hazards within the areas, however, the residents still experience hazard exposures due to the residents’ visitations to other direct exposure regions. Accordingly, the residents’ hazard exposure is termed as “latent exposure,” denoting that such exposure is not directly derived from their residential locale but their daily movement to other nonhome areas with high environmental hazards.

**Figure 2:**
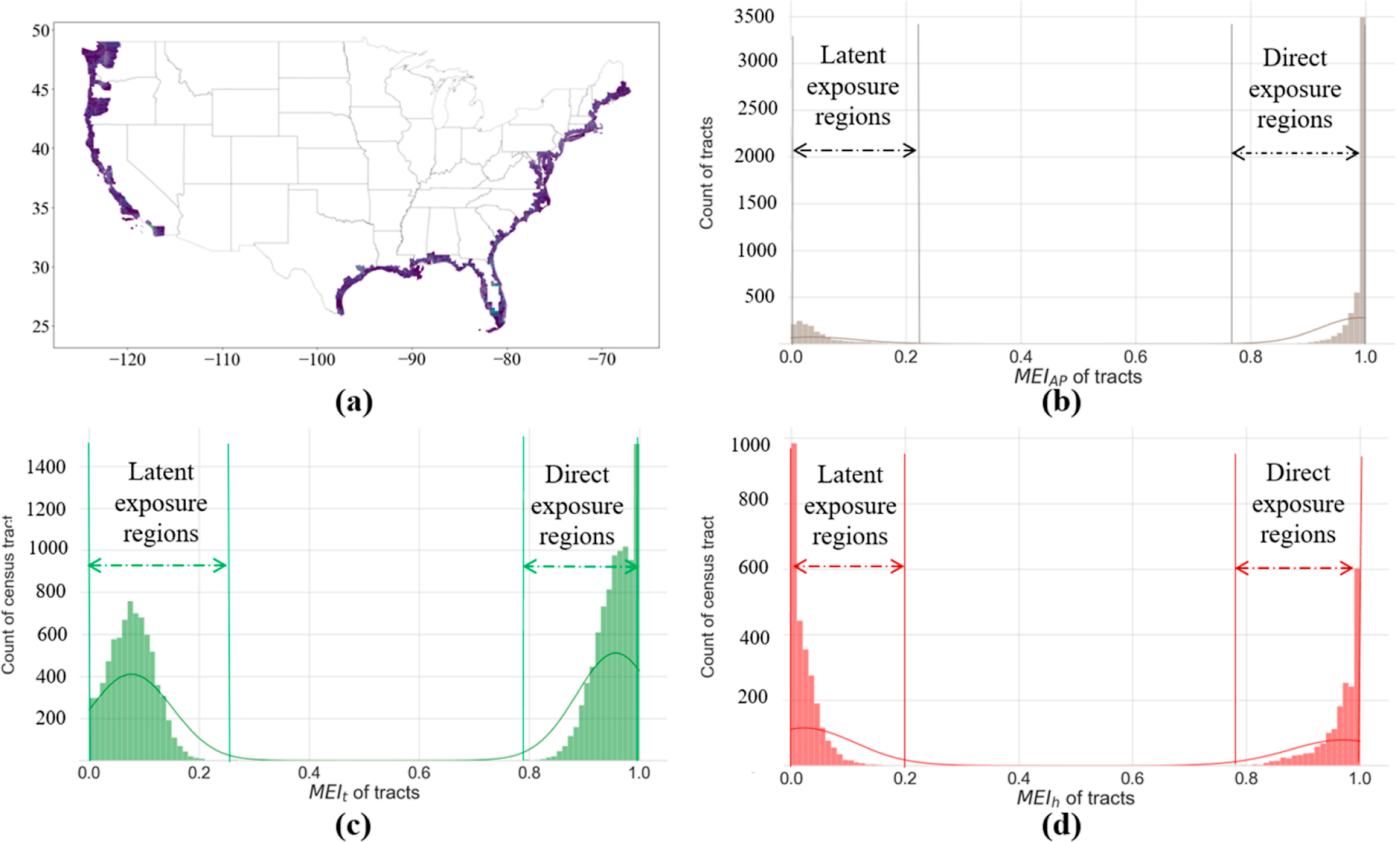
Distributions of MEI for different hazard types. (a) The geographical distributions of the study regions, along the coastal areas in the United States. (b–d) Histogram representation of the MEI_ap_, MEI_h_, and MEI_t_ across the study regions. For all the three studied hazards, the MEIs follow bimodal distribution with peaks at both ends, demonstrating distinct hazard exposures between direct and latent exposure regions and that residential locations are the major determinant for people’s hazard exposures. Note that tracts with no hazard exposure are excluded from the plot.

The distributions of MEIs show great unevenness across the studied region (Figure 2b–d). For all studied hazards (air pollution, toxic exposure, and heat), the MEIs show patterns of “bimodal distribution”, with MEI clustering around separate values and peaking at the two ends. Specifically, the direct exposure regions have higher exposure than the latent exposure regions. The mean values of MEI_ap_, MEI_t_, and MEI_h_ for the direct exposure regions are 98.9, 95.3, and 96.5%, as opposed to 4.4, 7.8, and 2.5% for the latent exposure regions, confirming the residential location as the major determinant for hazard exposures. The bimodal distribution MEI reveals the presence of a significant gap in the extent of environmental hazard exposure. One group of individuals spends more than 80% of their time in places with high-hazard areas, and another group spends less than 20% of their time in high-hazard areas. The presence of this significant divide in MEI is an indicator of environmental injustice and motivates additional analysis to examine the disparities for socially vulnerable populations.

**Figure 3:**
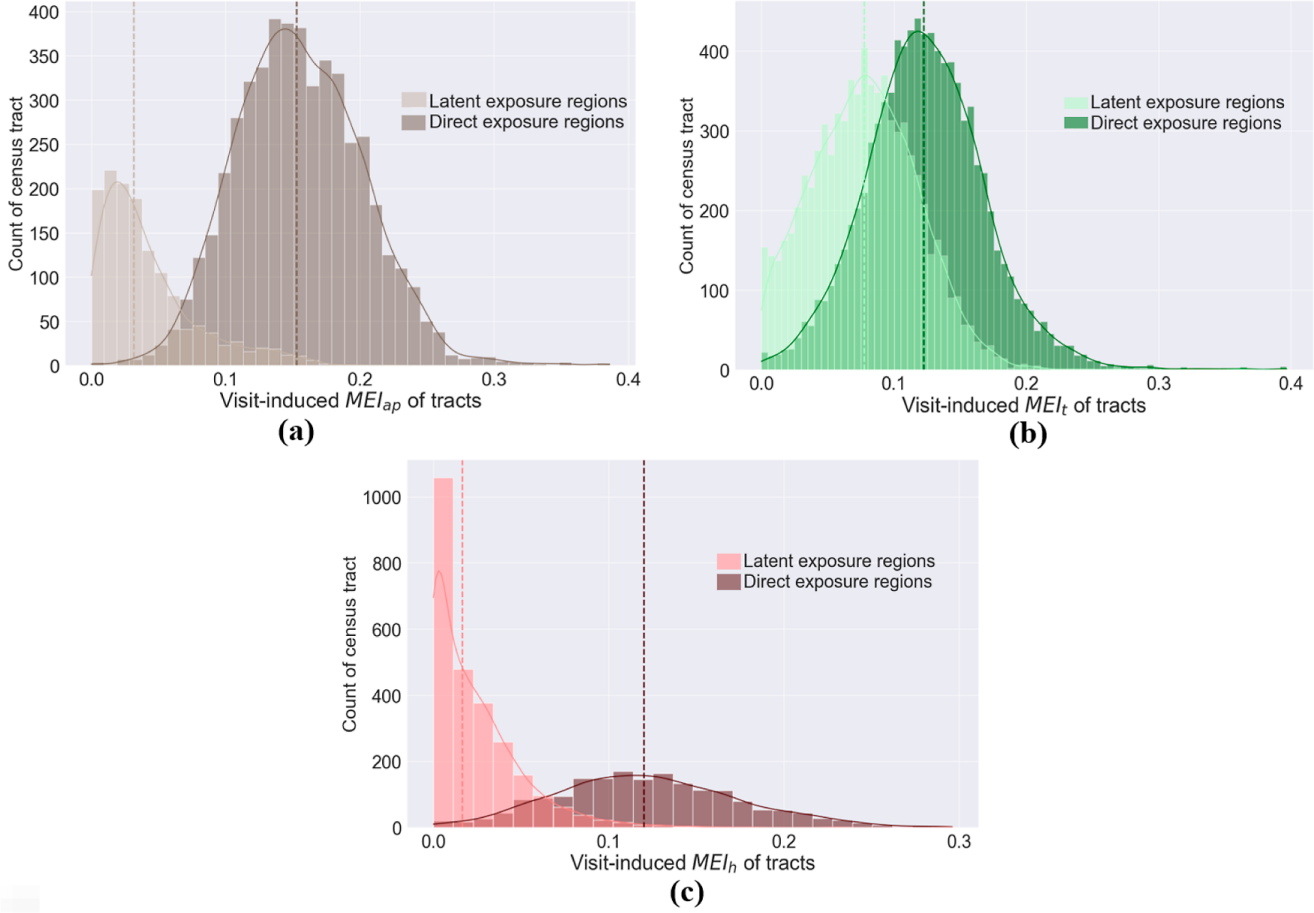
Comparisons of MEI due to populations’ visits to nonhome regions (i.e., other than their home census tract), between direct and latent exposure regions. Individuals residing in regions with direct hazard exposure experience greater exposure to hazards due to visits to nearby regions than individuals residing in areas with mere latent exposure.

To decipher the influence of mobility on hazard exposures, we further identify the hazard exposures induced by the residents’ visits to nonhome regions (the regions other than their home census tracts), for both direct and latent exposure regions (Figure 3). For the direct exposure regions, the mean values of traveling-induced MEI_ap_, MEI_t_, and MEI_h_ are 15.3, 12.2, and 12.0%, respectively, which are greater than those of the latent exposure regions, with increased MEI values of 3.2, 7.8, and 1.7%, respectively. These findings indicate that (1) individuals residing in regions with direct hazard exposure not only experience a greater degree of hazard exposure owing to their residence in a high-hazard census tract but also face an higher risk of exposure to hazards during visits to places in nearby high-hazard regions than individuals residing in areas with mere latent exposure; (2) disregarding the impact of mobility on environmental hazard exposure may result in the underestimation of hazard exposure risks, which is particularly evident in regions with direct exposure, where, on an average, more than 10% of hazard exposures may be underestimated. The fact that people residing in high-hazard areas have a greater exposure due to their mobility can be explained based on spatial clustering of hazards and laws of human mobility. According to the laws of human mobility, the frequency of visits to places has an inverse relationship with distance to home.^41^ Since environmental hazards are spatially clustered, a high-hazard census tract is likely to be surrounded by other high-hazard census tracts as well. Hence, when people visit places in nonhome census tracts, they are more likely to visit places in neighboring census tracts, which have similar levels of hazard exposure, more frequently.

**Figure 4:**
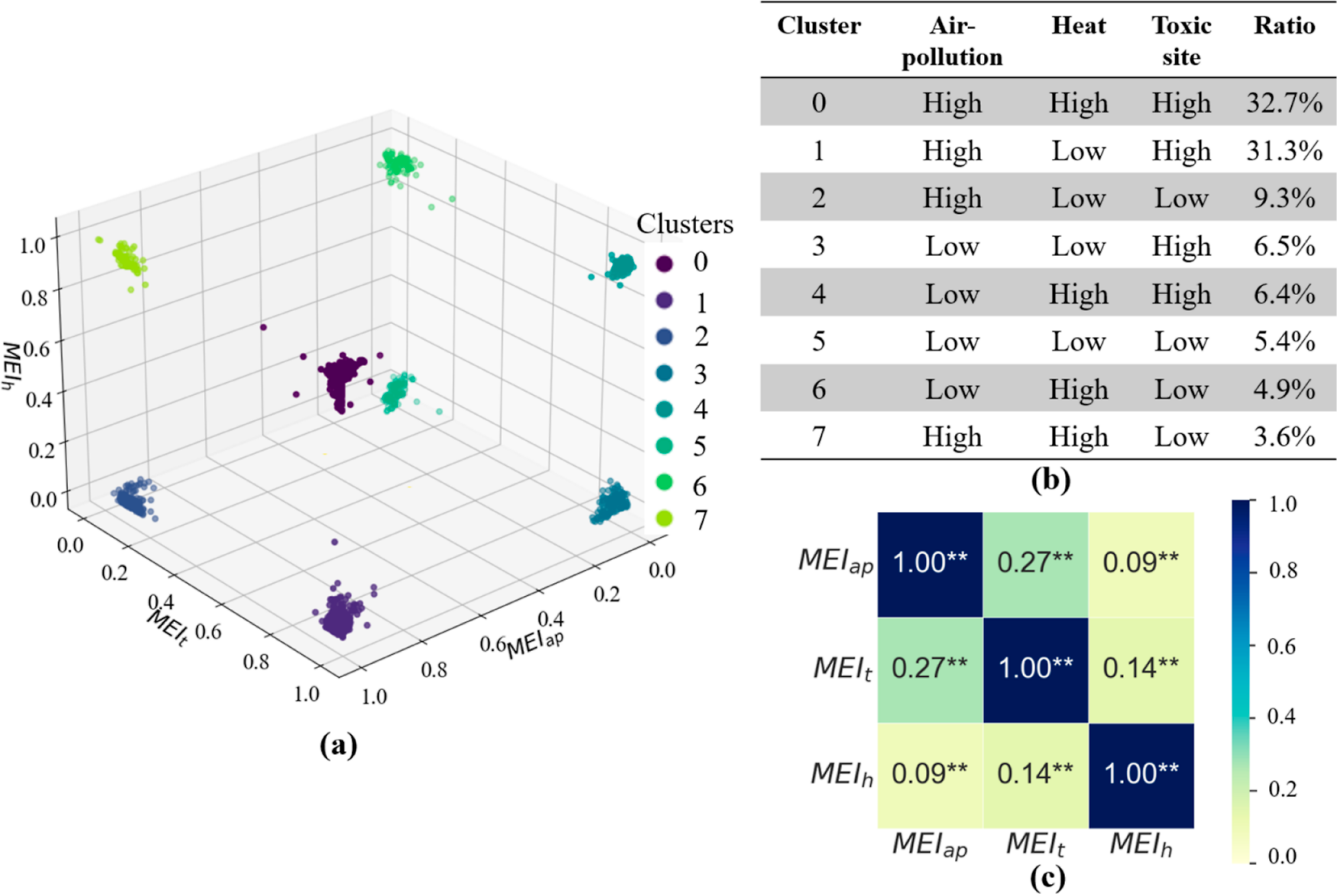
Classification of census tracts based on MEI patterns for all hazard types using DBSCAN. (a) Representation of the census tracts in three-dimensional space. Each point represents a census tract. The coordinates of the point are the tract’s possessed value of MEI_ap_, MEI_t_, and MEI_h_. The color indicates the tract’s category. (b) The count of tracts and occupation in respective hazards for each category. (c) Analysis of correlations between hazard pairs.

In addition, variations in the MEIs are evident across diverse geographical areas. As shown in Figure 4a,b, eight distinct clusters/categories have been identified according to the hazard exposures experienced by each tract (refer to Section 2.3).

This clustering analysis revealed that compounded environmental pressures are a common occurrence across the regions in our study. As shown in Figure 4, the exposure risks are far from homogeneous, painting a complex picture of the multihazard landscape. One category, in particular, stands out due to its high exposure to all the three types of hazards. This category, which makes up 32.7% of all tracts, underlines the severity of compound hazards. It indicates that nearly a third of the studied regions are grappling with the simultaneous impact of multiple hazards, adding layers of complexity to the mitigation efforts. A vast majority of the tracts (73.3%) experience high exposure to at least two types of hazards, further underscoring the pervasiveness of compound environmental pressures. This prevalence of multihazard exposure risks highlights the fact that for most regions, environmental hazards are not isolated issues, but interconnected problems that intensify each other’s impact. These findings emphasize the critical need for holistic, multihazard mitigation strategies that are capable of addressing the complexity of the environmental pressures faced by these regions.

Also, the co-occurrence of multiple types of hazards in specific areas suggests potential correlations between these hazards. Further analysis of the mobility-based hazard exposure index (Figure 4c) reveals significant positive correlations at a significance level of 0.01 between each pair of MEI_ap_, MEI_t_, and MEI_h_. This result reveals another important aspect of environmental injustice. People in high-hazard areas typically have high levels of exposure to two or more hazard types.

### 3.2 Latent Exposure Areas

**Figure 5:**
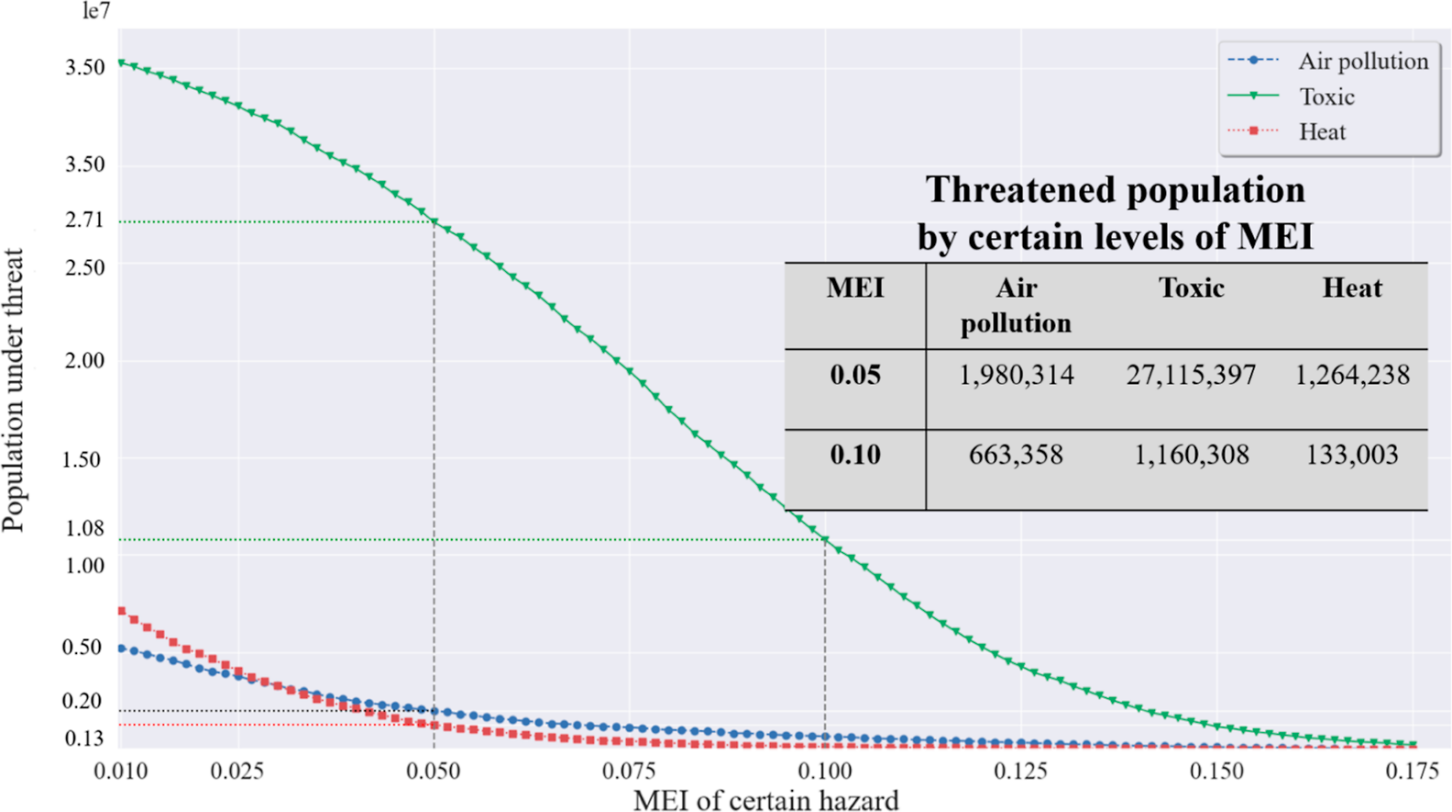
Populations affected by different hazard types in the latent exposure areas. The *x*-axis is the MEI, representing the proportions of the total time in high-hazard areas. The *y*-axis is the number of the affected population based on population census data at the census-tract level.

Individuals residing in regions without immediate hazard exposure may still encounter persistent and chronic hazard exposure due to their outbound visits to other high-hazard regions. Figure 5 plots affected populations that have certain levels of hazard exposure in the latent exposure areas. The results reveal that among the study areas (239 coastal counties in the United States), there are 1.16 million people who, despite residing in regions with no direct exposure to toxic sites, spend 10% of their time in areas where they are exposed to such sites due to their visits to other regions. In comparison, 0.13 million and 0.66 million individuals encounter similar degrees of latent exposure to air pollution and heat, respectively. When the threshold is set at 5%, populations with latent exposure to air pollution, heat, and toxic sites increase to 1.99 million, 1.26 million, and 27.11 million, respectively. While spending 5% of life activity times in high-hazard areas may not seem significant, persistent exposure for this duration could have dire health and well-being impacts. This result also highlights that exposures to toxic sites constitute the largest source of latent risk among the three types of investigated hazards.

**Figure 6:**
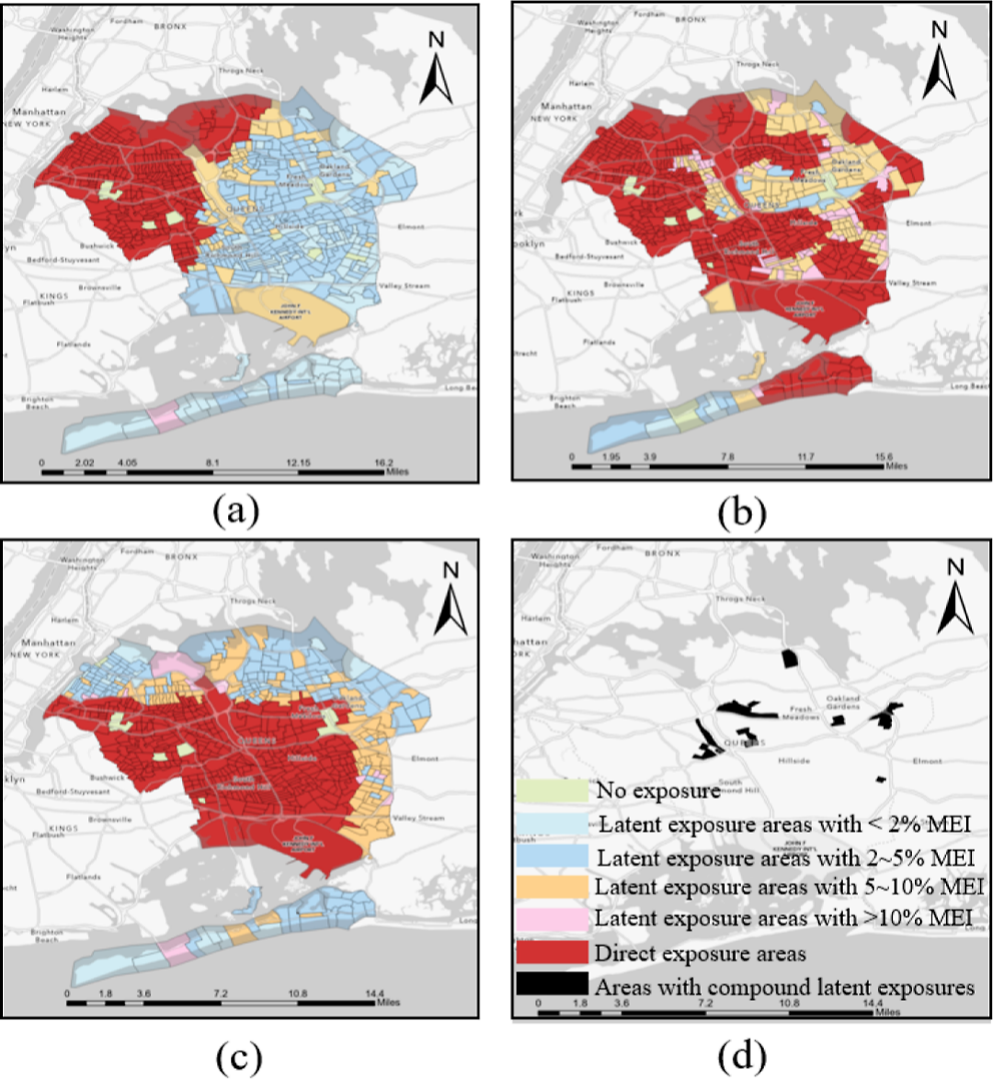
Various hazard exposures in Queens County in New York City, (a) air pollution, (b) toxic site exposure, (c) heat, and (d) compound (the areas affected by all the three hazards).

Some areas experience the combined effects of multiple latent hazard exposures. For example, among the 239 counties examined in our study, Queens County in New York City was identified as having census tracts with latent exposure to all three investigated hazards. Figure 6 depicts that over 54, 176, and 118 tracts in Queens County have an MEI_ap_, MEI_t_, and MEI_h_ exceeding 5%, which implies that residents in these areas are exposed to air pollution, toxic, and heat hazard exposures for more than 10% of their time due to travel to other high-hazard areas. Specifically, there are 19 tracts with latent exposure to all three hazard types, subjecting more than 60,000 persons to the compounded effects of these latent hazard exposures.

### 3.3 Disparity in Hazard Exposures

**Figure 7:**
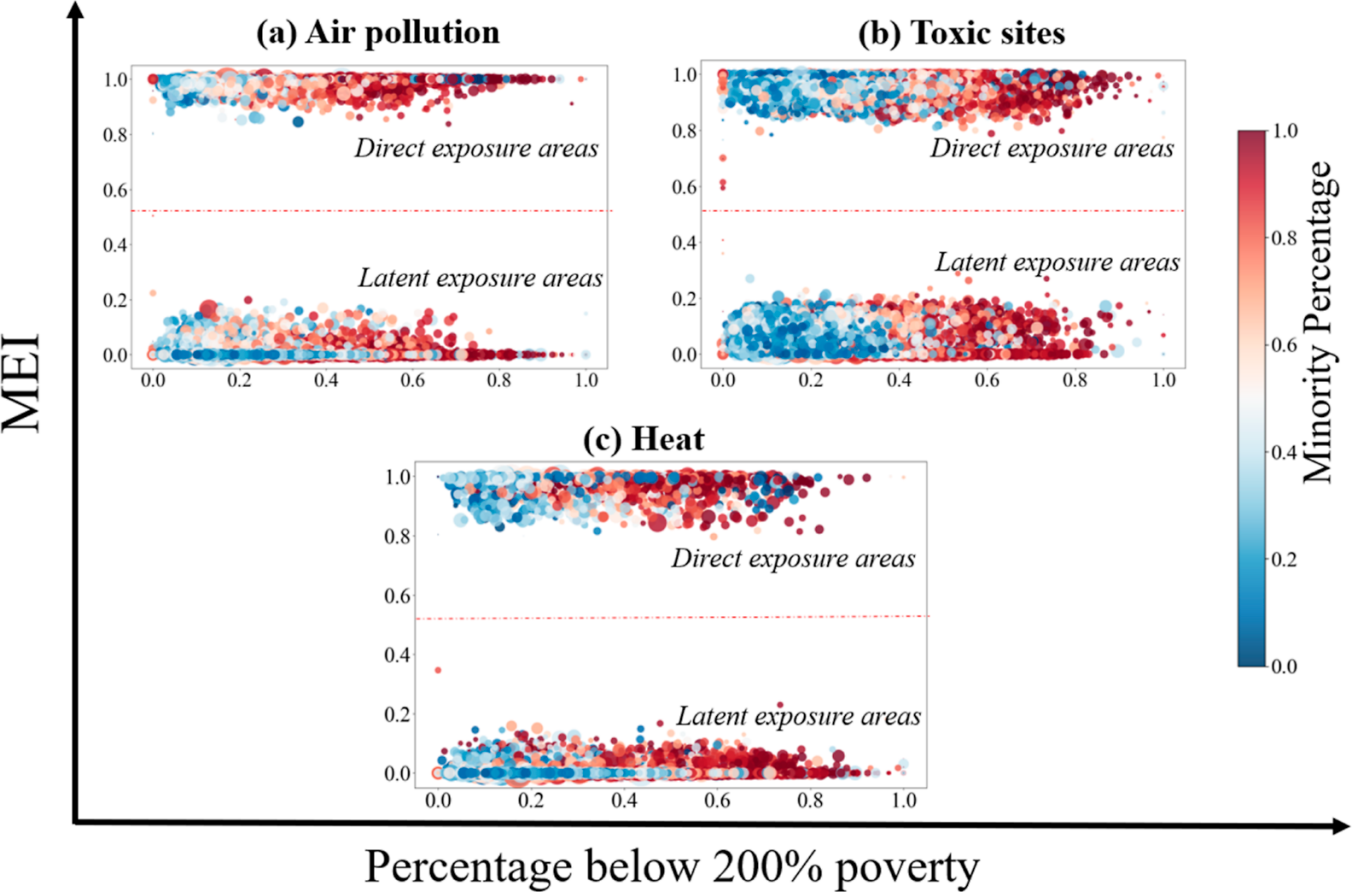
Hazard exposure (measured by MEI) versus community vulnerability indicator at the census tract-level for (a) air pollution, (b) toxic sites, and (c) heat. Each data point in the chart represents a tract. The *x*-axis represents the tract’s percentage of population below 200% poverty; the *y*-axis represents the tract’s the MEI for various hazards; the color of each point is rendered by the tract’s percentage of minority and the size of each point is scaled proportional to the tract’s population.

The results show significant disparity in MEI across different subpopulations, raising new concerns for environmental injustice. Figure 7 shows that, for all three hazard types, ethnic minority subpopulations and subpopulations with a greater degree of poverty have larger MEI values. A closer demographic comparison between the high-hazard exposure areas and other areas is shown in Table 1. The results show that, compared with the average level, both the direct and latent exposure areas generally have higher concentrations of a socially vulnerable community (i.e., minority and below-poverty population), with one exception for the latent air-pollution exposure areas. The results provide data-driven evidence that below poverty and ethnic minority residents have a greater mobility-based environmental hazard exposure (in both direct exposure areas and latent exposure areas).

**Table 1:** Statistics of Vulnerability Representativeness across Different Areas^a^

	average of all tracts (%)	direct exposure areas				latent exposure areas			
		air pollution (%)	toxic (%)	heat (%)	compound (%)	air pollution (%)	toxic (%)	heat (%)	compound (%)
percentage below 200% poverty	29.0	30.4**	31.8**	32.7**	35.9**	26.0**	33.8**	28.5**	53.4**
percentage of minority	49.8	58.0**	55.1**	58.4**	64.8**	48.9**	59.3**	53.9**	81.0**

a The numbers larger than the average of all tracts are in bold texts and ** represents *t*-test significant at 0.01 level.

Another noteworthy finding is that the areas exposed to all three hazards exhibit particularly high concentrations of vulnerable communities, with both below-poverty and minority populations being disproportionately represented. In areas with compound direct exposures, the below-poverty and minority populations account for 35.9 and 64.8% of the total population, respectively. For areas with compound latent exposures, these proportions rise even higher, to 53.4 and 81.0%. This indicates that vulnerable communities, especially those experiencing poverty and with minority populations, are disproportionately burdened by the compounding effects of multiple hazards. These findings highlight the urgent need for targeted interventions, policies, and resources that specifically address the unique challenges faced by these communities in order to reduce their vulnerability and promote environmental justice and equitable living conditions, which are further discussed.

## 4 Discussion

### 4.1 Spatial Autocorrelation and Human Mobility Potentially Create Traps in Direct Exposure Areas

The study areas in this work (the US coastal areas) have long been suffering from dire environmental hazards. For example, studies showed that coastal cities like Los Angeles, which recorded 157 unhealthy air days in 2020, are heavily impacted by air pollution.^42^ In fact, over 40% of Americans—about 135 million people—are exposed to unhealthy air levels, with a significant portion living in coastal regions. Concurrently, these areas are experiencing rising temperatures. Other studies indicated that over the past 50 years, US coastal regions are experiencing increasing warming.^43,44^ This has led to an increase in dangerously hot days, posing health risks to the population. In addition to heat and air pollution, the EPA also listed over 21,600 facilities across the United States as of 2021, many located in coastal states such as Texas, which alone accounted for over 1900 sites.^37^ The EPA’s Superfund Program has identified over 1300 sites, often found in coastal areas, representing a significant risk to nearby populations, potentially impacting millions of people.

Based on the identified environmental hazards across the study regions that are mentioned above, our results reveal a more nuanced understanding of the environmental risks endured by individuals living in areas with direct hazard exposure, showing that their exposure level extends beyond what was previously known and the risk from a seemingly insignificant exposure level could have dire health consequences. These individuals, while already susceptible due to residence in close proximity to hazard zones, face an additional risk as they visit places within nearby areas.

The observed increase in hazard exposure due to visits to places in nearby areas could be attributed to the spatial autocorrelation of high-hazard areas. Essentially, regions that are geographically close are more likely to exhibit similar hazard characteristics due to shared environmental and development factors and land use factors. For instance, air pollution levels might be high across a cluster of census tracts due to the prevalence of industries or dense traffic routes, not just in the immediate vicinity of an individual’s residence. Similarly, a region with high heat exposure due to the urban heat-island effect might be surrounded by areas with similar characteristics due to shared climatic conditions and urbanization patterns. This spatial autocorrelation means that individuals residing in regions with direct hazard exposure are likely to encounter similar, if not identical, hazards in their proximal surroundings when they travel.

Coupling with the spatial autocorrelation of hazards, human mobility law related to decay effect (i.e., “individuals are more likely to visit nearby regions”) is possibly another cause for accentuated traveling-induced hazard exposures.^45,46^ The human mobility patterns are greatly influenced by the urban environment and transportation.^30,47−49^ Given that people tend to frequent places that are closer to their residence, their mobility patterns essentially increase their dwell time in high-hazard environments if they live in high-risk areas. If their home and nearby regions share similar hazard characteristics due to spatial autocorrelation, their likelihood of exposure increases with each trip they make within this radius. This highlights the importance of taking into account both the geographical distribution of hazards and human mobility patterns when assessing the true extent of the hazard exposure risk. In other words, the combined effects of spatial autocorrelation of high-hazard areas and human mobility patterns create environmental hazard traps for residents living in these areas.

### 4.2 Complexity and Uncertainty in Estimating Individuals’ Hazard Exposure

The intricacies of estimating the toxic exposure warrant further scrutiny, particularly in accounting for the numerous uncertainties inherent in the process. For instance, daily commuting activities present an array of exposure possibilities. Urban environments, particularly bustling cities, are known to have heightened exposure areas. Therefore, people commuting into these areas ostensibly traverse zones of high exposure. However, the degree of their actual exposure might differ significantly based on the mode of transportation. Those enclosed in a vehicle with a robust air conditioning system that recirculates filtered air are effectively insulated from the direct impact of external environmental toxins. Their exposure level might be drastically reduced in comparison to their counterparts who commute on a bicycle or drive with their windows down and are directly exposed to air pollution. Thus, the medium and mode of transportation become critical factors that contribute to variability in exposure levels.

The discrepancies in exposure during commutes underline the need for more robust toxic exposure estimation models that incorporate these nuances and variabilities and extend beyond geographic locations and static environmental data, considering personal lifestyle choices, transportation preferences, and potentially the time of day when the commute occurs, as pollution levels vary throughout the day. As such, the current exposure estimation approaches may understate the actual risk for those who frequently pass through high exposure areas. This under-representation of exposure risk is a significant concern, particularly for the vulnerable groups who may lack access to air-conditioned vehicles and rely more on open-air transportation means. This necessitates more comprehensive exposure assessments that reflect the varied realities of daily life in urban environments.

### 4.3 Examining the Intersection of Hazards Interplay and Environmental Injustice

The findings from this study underline two significant aspects of environmental hazard exposure: the interrelated nature of different hazards and the disproportionate impact on vulnerable communities. First, the observed co-occurrence and significant positive correlations between the MEIs for the three hazard types suggest that these environmental threats interact and potentially exacerbate each other, creating a complex risk landscape. This interplay among air pollution, toxicity, and heat hazards could contribute to the heightened risk for individuals living in and visiting these high-hazard areas. Understanding this interrelation is critical, as it highlights the need for integrated environmental hazard mitigation strategies for tackling hazards rather than addressing them in isolation.

Second, the findings underscore a pressing environmental justice issue. First, the bimodal distribution of MEI values suggests a large divide in the extent of exposure of individuals for all three hazards types. This large divide provides data-driven evidence regarding the magnitude of environmental injustice in these counties. Second, it is alarming that below-poverty and minority populations are disproportionately represented in areas exposed to all three hazards. The higher representation of these subpopulations in areas with compound exposures is indicative of significant disparity in the distribution of environmental risks. These communities are shouldering an unjust burden, being more affected not only by individual hazards but also by the compound effects of all three hazards through both direct and latent exposures. This level of exposure exacerbates their existing vulnerability and poses significant challenges to their health and well-being. In light of these findings, it is crucial to emphasize the need for targeted interventions and policies that specifically address the unique challenges faced by these communities. Efforts should be made to reduce their vulnerability, promote environmental justice, and achieve equitable living conditions. This could involve, for instance, enhancing infrastructure in these areas to reduce hazard exposure, implementing stricter regulations on pollution sources, or providing better access to healthcare and resources for coping with hazards.^50,51^ Furthermore, these results underline the importance of incorporating an environmental justice lens in hazard management and urban planning policies to ensure a fair distribution of environmental risks

### 4.4 Exacerbated Hazard Exposures by Outbound Mobility

This study’s results have provided new insights into the role of human mobility in understanding and quantifying hazard exposure risks. Notably, the findings reveal a marked underestimation of these risks when mobility is not taken into account. Such a disregard can result in an over 10% underestimation of the hazard exposure risks. This underestimation is especially pronounced in regions characterized as direct exposure regions—areas where inhabitants face a high level of hazard exposure due to the very nature of their residential environments. These findings clearly demonstrate the limitations of conventional methods, which primarily focus on the residential location alone when assessing hazard exposure. Thus, this study underscores the need for a more nuanced approach that considers the impact of human mobility in these assessments.

Moreover, the findings shed light on the concept of latent exposure, which arises from visits to high-exposure zones outside residents’ home areas. Our analysis revealed that a significant portion of the population—running into millions—conduct 5 to 10% of their daily activities within high-exposure zones. This pattern is particularly striking, since it involves individuals residing in areas typically perceived as safe. It thereby reveals a layer of risk exposure that is usually overlooked: even though people might live in low-risk areas, their mobility patterns could lead to substantial hazard exposure. This exposure is not a one-time risk but recurs with every visit, thereby creating a continual risk scenario that can compound over time.

The observation of such extensive latent exposure significantly broadens the conventional understanding of hazard exposure. It goes beyond the static nature of residential location-based exposure and encompasses the dynamic nature of human mobility. This understanding emphasizes the need for a comprehensive, mobility-inclusive approach to assessing hazard exposure, one that takes into account both the places where people live and the places where they move in their daily lives. Given the scale of the population involved and the potential cumulative impact over time, the importance of including this broader perspective in hazard mitigation strategies and policy planning cannot be overemphasized.

### 4.5 Implications for Policy Making and Hazard Mitigations

The outcomes of this research have notable implications for policies aimed at mitigating environmental hazards. They accentuate the importance of factoring in human mobility data when planning and implementing these measures for a more holistic and precise understanding of exposure risks. By tracking and analyzing patterns of human movement, it is possible to identify and reduce latent exposures. For instance, in the case of air pollution hotspots, instead of solely focusing on residents in these areas, the mitigation strategies should also consider those who commute through these regions for work or school, thereby being exposed to the pollution during peak hours. By understanding that a large number of people commute from safer areas to high-exposure zones, policies could be developed to reduce the pollution levels in these zones or to develop infrastructure that minimizes exposure, such as sealed, air-filtered transport hubs. The resulting benefits would not be restricted to the residents of high-hazard areas but would extend to these commuters too, reducing their potential health impacts caused by routine visits to high-hazard regions.

Moreover, our findings are important complementation to information from established sources such as the Justice40 Initiative^52^ and EJScreen,^53^ and offer a more complete picture of risk exposure. Traditional methods largely focus on residential-based metrics, often overlooking the significant role of human mobility in the exposure risk. By integrating our mobility-based insights with these rich data sets, we have the opportunity to redefine how we identify and categorize high-risk areas. Our approach introduces a new layer to the analysis, accounting for the populations who may reside in lower-risk areas but frequently commute or travel through high-risk regions due to work or other life activities. Such a potential integration not only highlights census tracts or regions that may not have been previously recognized as high-risk using traditional residential-based metrics but also opens up the possibility of developing more inclusive and effective mitigation strategies. With this comprehensive and nuanced understanding of exposure risk, policies can be more precisely targeted, potentially expanding the scope of mitigation measures and ultimately promoting greater environmental justice.

### 4.6 Potential Bias Due to Data Collection

Due to the availability, the analyzed data set in this study is from the month of April 2019, which may cause certain data bias issue.

Human mobility patterns can show variations throughout the year, often influenced by a range of factors, such as seasonal weather variations, school and work schedules, public holidays, and cultural events, among others. These variations could lead to different exposure levels to environmental hazards at different times of the year. Furthermore, these patterns may differ from one location to another based on local contextual factors, which further adds to the complexity. Thus, the findings of our study, while providing valuable insights into the influence of human mobility on hazard exposure risks, may be influenced by the specificities of the data set in question. Consequently, this potential bias should be considered when interpreting our results. Future research in this area could benefit from a more longitudinal analysis that encompasses data from different periods of the year to account for seasonal variations in mobility patterns and their implications for hazard exposure.

In addition, it would be valuable to compare and contrast the mobility patterns and associated hazard exposure risks across different geographic locations. Such an approach would further elucidate the role of local contextual factors in shaping these patterns and risks, thereby contributing to a more nuanced understanding of the links between human mobility and environmental hazard exposure.

## 5 Contributions and Limitations

Environmental hazards pose significant threats to human health. Traditional approaches for hazard exposure evaluation have mostly focused on individuals’ home residence locations and neglect hazard exposure due to people’s mobility and dwell time in other regions. In this study, we create a mobility-based approach for quantifying the extent of environmental hazard exposure by utilizing a fine-grained large-scale human mobility data set.

The proposed mobility-based hazard exposure index and the findings from the analysis of 239 US coastal counties make multiple important contributions. First, by considering the extent of hazard exposure related to population activities and visits to places in other regions, this study provides a more reliable measure for quantifying environmental hazard exposures. The findings show that overlooking the influence of human movement on exposure to environmental hazards can potentially lead to underestimation of these risks. This issue is especially apparent in areas that face direct exposure, where on average there may be an underestimation of over 10% of hazard exposure instances. Second, the results showed that the combination of spatial clustering of high-hazard areas and distance-decay law of human mobility has led to creation of environmental hazard traps in which residents residing in high-hazard areas bear additional 10% exposure to environmental hazards due to their life activities and visitation to places in other areas.

Third, the findings reveal a significant divide in the extent of environmental hazard exposure in residents of communities. The bimodal distribution of MEI values for both direct exposure and latent exposure areas provide data-driven evidence for the severity of environmental injustice issues. In addition, the findings showed poor and ethnic minority residents are disproportionately exposed to all three environmental hazard types examined in this study. Also, these vulnerable populations are exposed to more than two environmental hazard types in most census tracts, which compounds the adverse health impacts of these hazards. These findings provide a deeper understanding of the extent of environmental injustice in US communities. Finally, the study enables the examination of latent exposure to environmental hazards caused by visits to places outside the home census tracts. The results show that millions of US residents in the counties studied spend 5–10% of their weekly life activities dwelling in places with high-hazard exposure. This latent exposure could compound over time and cause a chronic threat to the health of populations that are not living in high-hazard areas. A substantial portion of the population might be inadvertently exposed to various hazards due to their daily routines despite residing in areas perceived as safe. These findings offer important data-driven insights to public health officials, urban planners, and environmental policy makers regarding the extent of environmental hazard exposure in different areas of a community in formulating targeted policies to reduce the dire environmental injustice shown in this paper.

The results of this research suggest several worthwhile directions to explore in the future. First, the proposed approach in this work could be expanded to include other kinds of environmental hazards. By exploring more diverse hazards, researchers could provide a deeper understanding of the different ways in which mobility intersects with various forms of hazard exposure. Second, there lies an intriguing opportunity to model the relationship between mobility-based hazard exposure and certain health outcomes such as disease incidence, life expectancy, and mortality rates. A data-driven exploration of these relationships could yield invaluable insights into the public health implications of environmental hazards in conjunction with population activities and human mobility. Also, future studies could adopt the mobility-based approach proposed in this study to examine the effects of future urban development and climate change on population exposure to environmental hazards. For example, expansion of urban extents changes population density, modifies spatial distribution of hazards and alters human mobility.^54^ The analysis of the combined effects of city development and expansion using the mobility-based approach proposed in this study could inform integrated urban design strategies to address environmental hazards and public health impacts as cities continue to grow and expand. Finally, due to limitations in data availability, the focus of this study is restricted to US coastal areas. As further relevant data become accessible, future works can explore and examine the broader applicability and generalizability of our findings within a more extensive geographical context.

## Data Availability

The human mobility data sets were provided by Spectus Inc. The other data sets that support the findings of this study are available from upon reasonable request.
